# Preceptors training need assessment for medical laboratory professional clinical education programs in Ethiopia

**DOI:** 10.1371/journal.pone.0275533

**Published:** 2022-10-06

**Authors:** Abebe Edao Negesso, Gizachew Kedida Rikitu, Gelila Biresaw Sime, Endale H. Gebregzabher, Saba Gebremichael Tekele, Abay Sisay Misganaw

**Affiliations:** 1 Department of Medical Laboratory Sciences, College of Health Sciences, Addis Ababa University, Addis Ababa, Ethiopia; 2 Ethiopian Medical Laboratory Association, Addis Ababa, Ethiopia; 3 Department of Medical Laboratory Sciences, Arbaminch University, Arbaminch, Ethiopia; 4 Department of Biochemistry, St. Paul’s Hospital Millennium Medical College, Addis Ababa, Ethiopia; 5 Department of Medical Laboratory Sciences, College of Medicine and Health Sciences, Wollo University, Dessie, Ethiopia; AIIMS: All India Institute of Medical Sciences, INDIA

## Abstract

**Background:**

Health Workforce Improvement Program and professional associations recognized the need for a formalized method of providing academic education that would improve how preceptors teach and assess student. Thus, this study aimed to assess training needs of preceptors for Medical Laboratory Science clinical practicum education programs in Ethiopia.

**Methods:**

A cross-sectional survey design was implemented in targeted health facility throughout the country to assess academic educational needs of preceptors for Medical Laboratory clinical practicum education programs. The study participants were conveniently selected practicing health professionals who formally or informally nominated as a clinical trainer or acts as clinical trainer giving practical training to the student in the targeted practice setting. An adapted structured questionnaire modified to local context was used to conduct the survey and the perceived competency assessment used five scale of measurement (Not capable, Beginner, Advanced beginner, Competent, Proficient). The frequency was presented using tables and figures.

**Results:**

A total of 304 laboratory professionals participated in this study. More than half (52.6%) of the study participants were in the age group of 21 to 30 years and 264/304 (86.8%) were male. The majority (43.0%) of study participants had 6 to 10 years of experience and 212 (68.8%) did not receive clinical teaching skills training in the past two years. Regarding applying different hands-on teaching methods, the majority 38/304(12.5%) were not capable for role play and community based training, 49/304(16.1%) reported being Beginners, 85/304 (28%) said that they are advanced beginners in the competency. In this study, most study participants 98/304(32.2%) and 130/304(42.8%) perceived that they are competent and proficient in applying laboratory practice teaching methods respectively.

**Conclusion:**

The average cumulative level of competency from level 1 (not capable) to level 3 (advanced beginner), we found: learning in the practical teaching area 45.4%, clinical practicum teaching quality improvement and advocacy 42.9%, student assessment methods 42.7%, communication, collaboration and partnership 40.9%. Overall competence of preceptors (proportion of preceptors reported competent) was 57%. We recommend designing the performance interventions in the form of training by including communication skills for effective preceptor ship, students assessment and feedback, teaching and instruction strategies, planning for clinical practicum learning and principles of learning and teaching in practical areas.

## Background

The quality of clinical education given in the practicing sites is important factors in order to enable students learn competencies in a standardized way and develop a good attitude towards the role they are going to assume in their future career. Clinical education is a teaching learning process which takes place in a real clinical environment where there are patients, preceptors, students and their care takers. The clinical component of a medical education program is arguably the most important part of the student education. It is a golden opportunity of practice as part of a continuum of learning from classroom teaching and simulation in a skill development laboratory. Through this experience, students gain & integrate essential knowledge, skills, values and become socialized into the profession. The most important factor for clinical teaching is preceptor [1, 2].

A preceptor is defined as a tutor who gives personal instruction, training, and supervision to the student in the practice setting [3, 4]. Preceptors facilitate the development of knowledge, clinical skills and professional attributes in health science fields. Preceptors encourage the enhancement of critical thinking and problem-solving skills that are trademarks of an academic education [5]. A preceptor is both a clinical teacher and a practicing health professional in a work setting; he/she guides students (or new graduates) in learning how to apply theoretical knowledge in to practice. They are important in the education and socialization process of students [2].

The learning partnership is a collaborative relationship between clinical setting and an academic institution, involves three key participants: preceptee, preceptor, and faculty advisor. The patient/ family is at the center of this relationship, as their health and safety is a primary concern. The stakeholders, preceptee, preceptor, faculty advisor, clinical facility and academic institution need to share mutual trust and respect and are expected to make a strong commitment to a positive learning experience [1]. The preceptorship program bridges the gap between the classroom teaching and clinical area practices. Although many health care practitioners have excellent clinical skills, their roles in teaching students are less refined. Therefore, it is essential to have health professional preceptors’ programs that strengthen their teaching competencies [6].

Studies have showed that preceptorship is negatively affected by the preceptors’ perceived insufficient knowledge and skills to meet the preceptees’ learning outcomes [7]. To ensure a consistent approach to every preceptorship experience, preceptor teaching skill competency should be measured or assessed. Competency for preceptor is the application of knowledge, skills, and abilities needed to fulfill organizational, departmental, and practice setting requirements under the varied circumstances of real-world situations. The goals of competency assessment of preceptors is to provide a mechanism for directing and evaluating the competencies needed by preceptee to provide quality healthcare services, to identify areas of growth and development and to provide opportunities for ongoing learning to achieve continuous quality improvement [8].

## Methods

### Study design, study settings and data collection period

A cross-sectional study was conducted from August 2 to August 22, 2021 on selected Medical Laboratory science clinical preceptors to assess their clinical teaching and assessment skills. The study setting was the public health facilities that are used by higher education institutions as a clinical attachment site/ industry across the nation. This includes teaching hospital, referral hospital, primary hospital, and regional hospital and health centers.

### Study population

The study population was clinical preceptors in medical laboratory science department; who formally or informally nominated as a preceptor or act as a preceptor working in teaching hospitals, referral hospitals, general hospitals, primary hospitals, health centers, and any other health care delivery system. They may have formal pre-preceptor training, or without prior knowledge and practice of preceptorship was the target population.

### Eligibility criteria

Medical laboratory professionals who have been working in that facility as a clinical practicum preceptor for a minimum of 6 months were eligible for the study. Those medical laboratory professionals who were part-time workers or have less than 6 months of experience in that hospital or no involvement in teaching students or not willing to take part in the study were excluded from the study.

### Sample size and sampling technique

This need assessment survey used convenience sampling technique to select survey participants. First, the health facilities (under the 4 regions and 2 city administrations) that were used actively for clinical teaching purposes were identified by the Ethiopian Medical Laboratory Association (EMLA) through a preliminary rapid assessment. Then, proportionate quota was allocated for regions and city administrations while health facilities beneath them were selected purposefully by ensuring reasonable representations in type of facility (health center, primary, secondary and tertiary hospitals ect). All the clinical preceptors in the medical laboratory science found in the selected health facilities were part of this survey. The sample size for this survey was calculated using single population formula as follows:

$$\text{n}=\frac{{\left({\text{Z}}_{\text{a}/2}\right)}^{2}\text{P}\left(1-\text{P}\right)}{{\text{d}}^{2}}$$

Where: n = sample size; Z = the standardized normal distribution curve value for the 95% confidence interval (1.96); P = proportion of population by using the previously reported overall competence of preceptors of 46% [9], d = margin of error which will be taken as 5%; and a 10% non-response rate was added to the calculated sample size. We assume our total population (N) from which were sampling was 2000. Therefore, the calculated sample size was 321 and considering 10% nonresponse rate sample size was = 321+32 = 353.

### Data collection tools and procedures

A structured questionnaire was used to assess clinical teaching and assessment skills of preceptors. Validation of the tool was done in two phases aiming to assess the capability of instrument to measure pedagogical competency of the preceptors in the view of specialists. The focused group discussion from subject matter experts of medical laboratory professionals evaluated whether the items in the scale represent the domains or concepts of preceptor competency assessment on the actual ground. The second phase validation used the data collection tool to assess the teaching and assessment skill of actual preceptor through pilot study. The Pilot study also aimed to measure inter-rater/intra rater reliability of the items and measurement scales in the tool, and to develop the sampling frame from each hospital nationally. The data for the pilot study was collected in the same way with self-administered questionnaire sent to the preceptors through email.

Data was collected using self-administered Google form questionnaires which consists of the seven components; Background information; Learning in the clinical area; communication, collaboration, and partnership; student assessment; clinical teaching quality improvement and advocacy; challenges and solutions and specific training needs.

The perceived competency assessment used the following scale of measurement 1 (Not capable): requires training and supervision before performing the task, 2 (Beginner): May perform the task but direct supervision needed at all times, 3 (Advanced beginner): Able to perform the task routinely under indirect supervision, 4 (Competent): Able to perform the task routinely without the need for supervision, 5 (Proficient): Able to perform the task routinely, even in complex contexts, and can train and supervise other teachers/ preceptors.

### Ethical considerations

Ethical clearance was obtained from the ethical clearance committee of Yekatit 12 hospital before the start of the study. The purposes and the importance of the study was explained & informed consent was obtained from each participant by the data collector. Confidentiality was maintained at all levels of the study by avoiding identifiers and using codes to identify preceptors. Participant’s involvement in the study was on voluntary bases.

## Results

### Socio-demographic characteristics of study participants

A total of 304 laboratory professionals participated in this study with a response rate of 86% among 353 target groups from 4 regional governments and 2 city administrations with a median age group of 21 to 50 years, with a median age of 30 years. The training health institutions were encompass 30.3% teaching hospital, followed by general hospital (19.1%). More than half (52.6%) of the study participants were in the age group of 21 to 30 years and 264/304 (86.8%) were male. Among study participants, the majority (43.0%) of study participants had 6 to 10 years of experience and 212/304(68.8%) did not receive clinical/practicum teaching skills training in the past two years.

Regarding the type of training institution, most (30.3%) students came for clinical laboratory practice to teaching hospital. About 239/304(78.6%) of study participants know their role and responsibilities as a trainer/preceptor and most 256/304(84.2%) study participants prefer Hands on training type of capacity building mode of delivery (Table 1).

**Table 1:** Socio demographic characteristics of the study participants in preceptors for Medical Laboratory Science clinical practicum education programs in Ethiopia, 2021(N = 304).

Variable	Frequency	Percentage
**Age (years)**		
21–30	160	52.6
31–40	125	41.2
>40	19	6.6
**Sex**		
Male	264	86.8
Female	40	13.2
**Region**		
Oromia	83	27.3
Amhara	72	23.7
Sidama	21	6.9
Diredawa	14	4.6
Addis Ababa	43	14.1
SNNP	71	23.4
**Level of Education**		
Diploma	33	10.9
BSc	181	59.5
MSc	85	28.0
Other	5	1.6
**Year of experience**		
1–5	87	28.5
6–10	131	43.0
11–15	50	16.4
15–20	22	7.2
>20	6	1.9
**Type of training institution where the students came for clinical laboratory practice**		
Teaching hospitals	92	30.3
Referral Hospitals	39	12.8
General Hospitals	58	19.1
Primary Hospitals	22	7.2
Health centers	46	15.1
Government Research Laboratories	20	6.6
Other	27	8.8
**Clinical/practicum teaching skills training in the past two years**		
Yes	92	30.3
No	212	68.8
**Role and responsibilities as a trainer/preceptor**		
Yes	239	78.6
No	65	21.4
**Capacity building mode of delivery do you prefer**		
Hands on training	256	84.2
Virtual	36	11.8
Other	12	3.9
**Computer access**		
Yes	68	22.4
No	236	77.6
**Internet access**		
Yes	66	21.7
No	238	78.3

### Learning in the practical teaching area

When we see the preceptors perceived level of competency on the skill of developing clear and measurable learning objectives for practicum/clinical sessions, 48/304 (15.8%) reported being Not capable, which indicates requiring training and supervision before performing the task. The majority 63/304 (20.7%) perceived that they are beginners in developing plan for clinical teaching sessions meaning that they are able to perform the task under direct or indirect supervision. About 77/304 (25.3%) of study participants were advanced beginner for creating opportunities for learners to develop critical thinking and reasoning skills. Among the study participants, 98/304 (32.2%) reported being competent in creating and maintaining a safe practical teaching environment for the students that is free from physical, psychological or any other form of harm leading to the interpretation that they are able to perform the task routinely without the need for supervision and the remaining 93/304 (30.6%) were proficient enough being able to perform the task routinely, even in complex contexts, and can train and supervise other teachers/ preceptors (Table 2).

**Table 2:** Learning in the practical teaching area of preceptors for Medical Laboratory Science clinical practicum education programs in Ethiopia, 2021(N = 304).

Statements	Not capable n (%)	Beginner n (%)	Advanced beginner n (%)	Competent n (%)	Proficient n (%)
Develop clear and measurable learning objectives for practicum/clinical sessions	48(15.8)	53(17.4)	59(19.4)	89(29.3)	55(18.1)
Develop plan for practicum/clinical teaching sessions	36(11.8)	63(20.7)	67(22)	75(24.7)	63(20.7)
Obtain informed consent for student involvement in actual practice	28(9.2)	54(17.8)	58(19.1)	83(27.3)	81(26.6)
Select appropriate teaching and learning materials and resources that are matched to the learning outcomes.	29(9.5)	41(13.5)	70(23)	89(29.3)	75(24.7)
Create opportunities for learners to develop critical thinking and reasoning skills	31(10.2)	32(10.5)	77(25.3)	89(29.3)	75(24.7)
Create and maintain a safe practical teaching environment for the students that is free from physical, psychological or any other form of harm	26(8.6)	32(10.5)	55(18.1)	98(32.2)	93(30.6)
Engage students in critical and constructive self-assessment skill and peer feedback	31(10.2)	36(11.8)	73(24)	86(28.3)	78(25.7)
Demonstrate the principles of effective delegation and supervision to students	28(9.2)	27(8.9)	75(24.7)	89(29.3)	85(28)
Use open educational (updated guidelines, protocols, research findings, digital learning platforms, etc) resources effectively	32(10.5)	39(12.8)	64(21.1)	89(29.3)	80(26.3)

### Applying different hands-on teaching strategies

**Fig 1:**
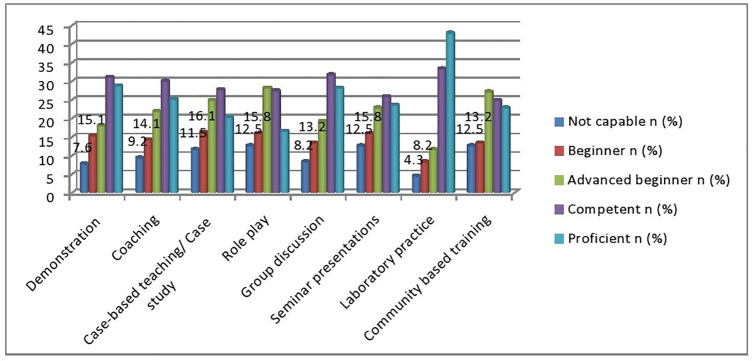
Apply different hands-on teaching strategies/ methods that are matched to the learning outcomes of preceptors for Medical Laboratory Science clinical practicum education programs in Ethiopia, 2021(N = 304).

Analysis of the data showed that the respondent’s response in applying different hands-on teaching strategies/methods that are matched to the learning outcomes, the majority 38/304(12.5%) were not capable for role play and community based training, 49/304(16.1%) reported being Beginners, 85/304 (28%) said that they are advanced beginners in the competency. In this study, most study participants 98/304(32.2%) and 130/304(42.8%) perceived that they are competent and proficient in applying laboratory practice teaching strategies/ methods respectively (Fig 1).

### Applying principles of gender responsive pedagogy

**Fig 2:**
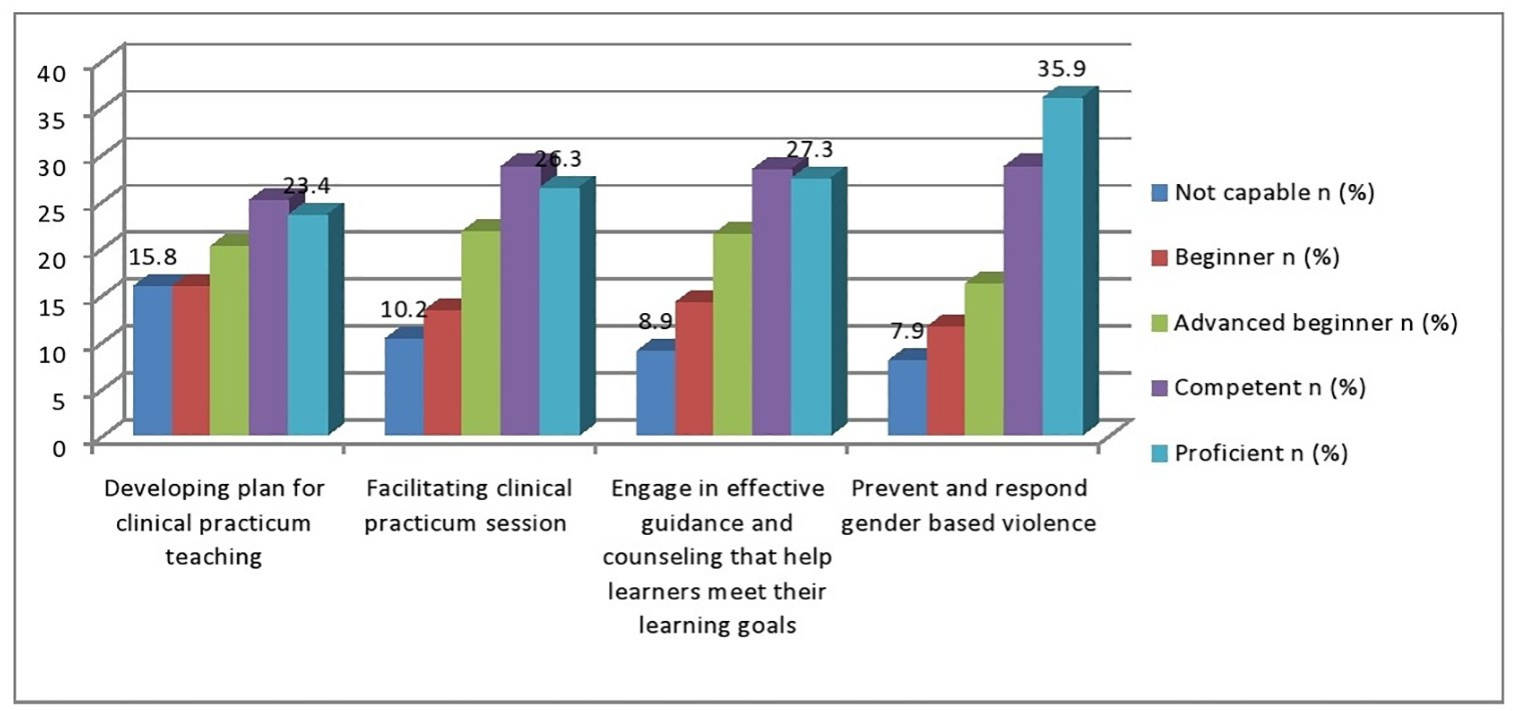
Apply principles of gender responsive pedagogy of preceptors for Medical Laboratory Science clinical practicum education programs in Ethiopia, 2021(N = 304).

In this study, the majority of study participants 48/304(15.8%) responded that they are not capable in applying principles of gender responsive pedagogy in developing plan for clinical practicum teaching. Moreover, significant number of study participants, 109/304(35.9%) answered that they are proficient in preventing and responding gender based violence (Fig 2).

### Communication, collaboration and partnership

The analysis result in the use of communication, collaboration and partnership that are matched with foster teamwork and collaboration between academic and practical environment, show that quite a majority of the respondents are competent 114/304(37.5%). Whereas 78(25.7%) of participants reported that they are proficient in teaching students on how to address patients with special need. About29/304(9.5%), 41/304(13.5%) and 78(25.7) of our participating preceptors are in the not capable, beginner and advanced beginner state respectively, indicating an area of intervention (Table 3).

**Table 3:** Communication, collaboration and partnership of preceptors for Medical Laboratory Science clinical practicum education programs in Ethiopia, 2021(N = 304).

Statements	Not capable n (%)	Beginner n (%)	Advanced beginner n (%)	Competent n (%)	Proficient n (%)
Communicate best practice in practical education with peers, students and other stakeholders	19(6.3)	41(13.5)	64(21.1)	106(34.9)	74(24.3)
Foster teamwork and collaboration between academic and practical environment	24(7.9)	24(7.9)	71(23.4)	114(37.5)	71(23.4)
Build and maintain collegial relationships and collaborations with multidisciplinary staff in the practical practicum teaching environment	22(7.2)	27(8.9)	78(25.7)	105(34.5)	72(23.7)
Use oral, written and electronic communication in practical teaching in order to achieve learner outcomes	18(5.9)	34(11.2)	76(25)	105(34.5)	71(23.4)
Teach students on how to address patients with special need	25(8.2)	40(13.2)	60(19.7)	101(33.2)	78(25.7)
Teach students how to provide health education to patients and their families/ caregiver.	29(9.5)	40(13.2)	53(17.4)	109(35.9)	73(24)

### Student assessment

Student assessment is the process of evaluating students’ abilities and achievements. It is actually encompasses a variety of ways to determine how students are progressing in their learning. From the study participants to assess educational needs of preceptors for Medical Laboratory Science clinical practicum education programs in Ethiopia. A higher percentage of study participants79 (26%) were proficient in providing timely constructive feedback to students and 113 (37.2%) were competent for individual oral interview/exam/Viva. In response to objectively structured practical exams (OSPEs), 39 (12.8%) of the study participants responded that they are not capable for such method of assessment (Table 4).

**Table 4:** Student assessment method of preceptors for Medical Laboratory Science clinical practicum education programs in Ethiopia, 2021(N = 304).

Statements	Not capable n (%)	Beginner n (%)	Advanced beginner n (%)	Competent n (%)	Proficient n (%)
Select and use assessment methods that are matched with the learning outcomes	34(12.1)	39(12.8)	69(22.7)	106(34.9)	56(18.4)
Workplace based assessments	21(6.9)	36(11.6)	69(22.7)	104(34.2)	74(24.3)
Individual oral interview/exam/Viva	28(9.2)	37(12.2)	67(22)	113(37.2)	59(19.4)
Objectively structured practical exams (OSPEs)	39(12.8)	28(9.2)	67(22)	105(34.5)	65(21.4)
Performance/ procedural checklist	28(9.2)	32(10.5)	69(22.7)	107(35.2)	68(22.4)
Use assessment data to enhance the practical teaching-learning process	29(9.5)	39(12.8)	65(21.4)	105(34.5)	66(21.7)
Maintain accurate records of student progress	25(8.3)	29(9.5)	65(21.4)	107(35.2)	75(24.9)
Provide timely constructive feedback to students	20(6.6)	32(10.5)	70(23)	103(33.9)	79(26)

### Clinical practicum teaching quality improvement and advocacy

**Fig 3:**
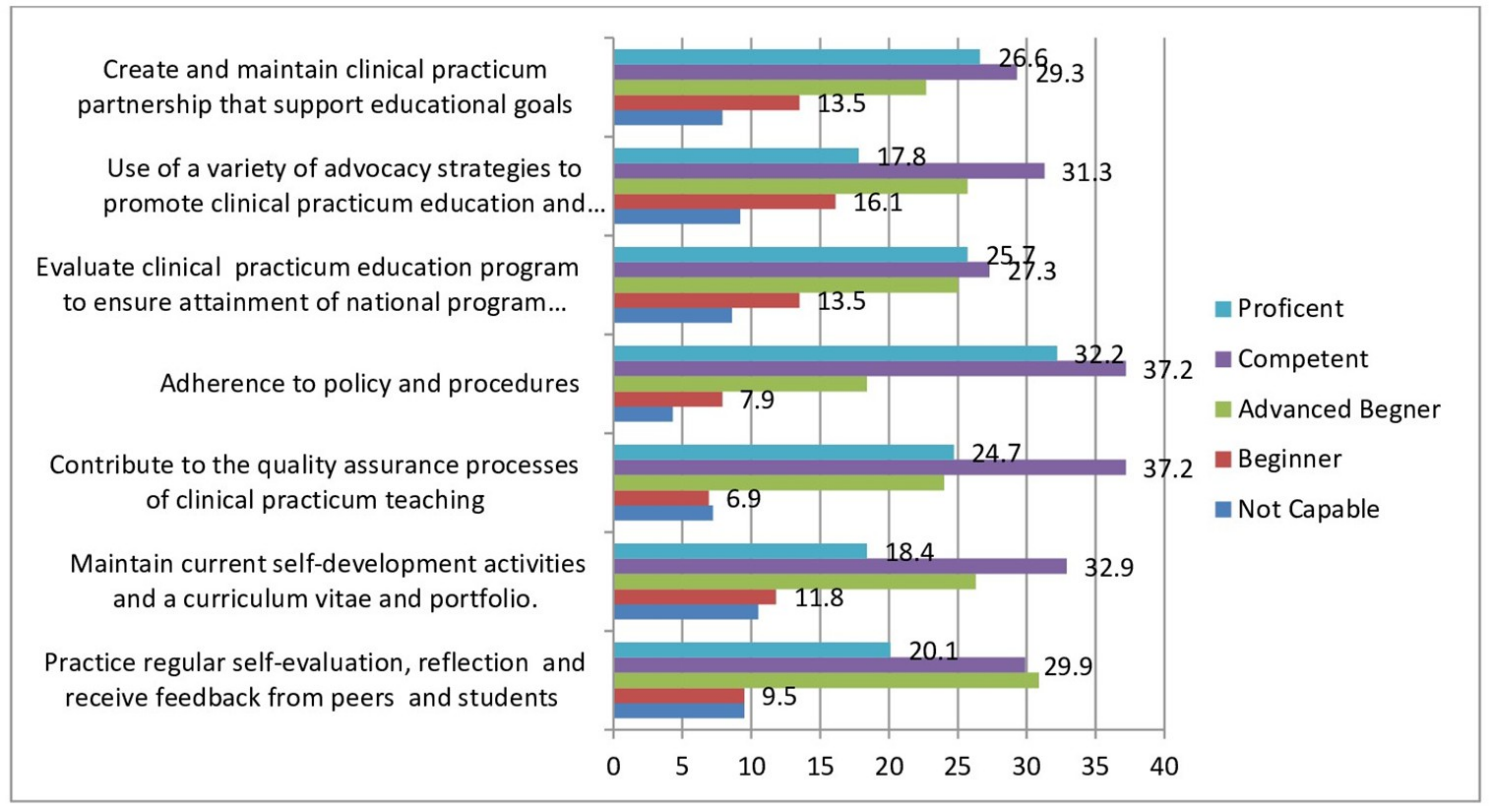
Clinical practicum teaching quality improvement and advocacy of preceptors for Medical Laboratory Science clinical practicum education programs in Ethiopia, 2021(N = 304).

From the existing collected data, we found that 30.9% were advance beginner against the practices regular self-evaluation, reflection and receive feedback from peers and students which means able to perform the task under direct or indirect supervision and also around 10% of them were not capable in maintaining current self-development activities and a curriculum vitae and portfolio which requires training and supervision before performing the task. When we see adherence to policy and procedures requirement 32.2% of the preceptors were able to perform the task routinely, even in complex contexts, and can train and supervise other teachers/ preceptors and 37.2% of the preceptors were contribute to the quality assurance processes of clinical practicum teaching (Fig 3).

Generally, based on our finding among the four domains, the average cumulative level of competency from level 1 (not capable) to level 3 (advanced beginner), we found: Learning in the practical teaching area 45.4%, Clinical practicum teaching quality improvement and Advocacy 42.9%, Student Assessment methods 42.7%, Communication, Collaboration and Partnership 40.9%. Overall competence of preceptors (proportion of preceptors who reported as at least competent) was 57%.

## Discussion

Preceptors training need assessment helps to have appropriate credentials for the practice setting, showing features of professionalism and mentorship for patients. In turn this plays important role in the production of knowledgeable, skilled and competent graduates. Moreover, preceptors training need assessment enables students to develop a good attitude towards the role they are going to assume in their future career and most preceptors are serving as role models in their particular setting for students [10].

According to the present study, the minimum educational qualification of a preceptor was Bachelor’s degree (59.5%). This was in line with finding which showed the majority of preceptors had a Bachelor’s degree [11–13]. Even though, the majority of preceptors in this study having degree level education, they were challenged with the level of competency. This finding differs from study indicated that preceptors prepared at degree level had good understanding associated with the role of educator [14].

In addition to educational level, a clinical experience of two years or more was selected as the minimum experience a preceptor should have which was supported by reports indicating a minimum of a baccalaureate degree and two years of experience as the two most chosen responses for selection of preceptors [15]. Therefore, these results emphasizes that preceptors should be clinically skillful and be supported by theoretical knowledge.

In the present study, 68.8% of study participants do not train for clinical teaching skills. This finding was agreed with the study reported that most (72%) of respondents do not train due to different barriers. Among these, lack of access to a preceptor-training program was the major barrier that participants had experienced [16]. In contrast, Andreas and Daniel reported more than half (56.8%) of preceptors knowing about clinical preceptorship using training and workshops [17]. Other researchers also highlighted that on-going preceptor training has impact on preceptorship skills [18–20]. However, there are differences in the content and duration of the existing preceptor-training programs, majority of study agreed that training for clinical/practicum teaching skill is a major way of acquiring preceptorship knowledge.

Preceporship is a cooperative process between academia and practice. Therefore, preceptors are required to use the same methods in their own teaching by practicing teaching principles [15, 21] and they also claimed the need for some understanding of how to teach students but according to different studies [22, 23], preceptors first need to learn how to identify needs and weaknesses of learner’s to ensure the learner needs before using new teaching and learning methods.

Findings from the current study revealed that 78.6% of participants recognized their role and responsibilities as a laboratory trainer/preceptor which was in contrast with study reported in Ethiopia 82.6% of respondent are not familiar with the concept of nursing clinical preceptorship [24] and another study in Nigeria, found that 65% of respondents are not knowledgeable towards Evidence-based Practice [25].

Communication described as one of the criteria to measure the competency of preceptors. Hence, listening, feedback, evaluation and interpersonal skills were listed as good communication [26]. The use of communication, collaboration and partnership that are matched with foster teamwork and collaboration between academic and practical environment, show that quite a majority of the respondents are competent in the present study. This finding was agreed with the report indicating good communication skill is one of the characteristics of an effective preceptor and contributed to 50% of all responses which are among the most important competencies of preceptors [22, 27].

Assessment should be the platform for an open and transparent feedback discussion between the student and the preceptor on progress in clinical learning [25]. This kind of feedback can enable students to set clear learning objectives and help them to take ownership of their learning and become more self-monitoring [17].

Regarding student assessment, current study indicated that 26% of study participants were proficient in providing timely constructive feedback to students and 37.2% were competent for individual oral interview/exam/Viva. In contrast, Green and colleagues [28] indicate that the use of a portfolio can facilitate reflection, provide evidence of knowledge, skills and experience. Other researchers indicated that precepting is depend on the feedback given to be able to take ownership of their learning which showed constructive feedback is crucial and important for learning [29].

The definition competency dependes on the level of professional education and experience of healthcare provider. Competency means essential skill of a person related to job performance [30] or it can be defined as the context of particular knowledge, attitude, and skill to perform a specific task which can yields desirable outcomes [31].

The overall competence of preceptors in the present study was 57% which was in close agreement with study in Northwest Ethiopia reported 48.7% of the study participants perceived themselves as competent [32] and 47.8% of study participants have high clinical competency [32]. Some studies tried to show the direct relation between competency of preceptors and competency of students. These competency of preceptors helps to increase the overall competency of students and also provide a mechanism for evaluating the competency of students.

## Conclusions & recommendations

The current study aimed to assess educational needs of preceptors for Medical Laboratory Science clinical practicum education programs in Ethiopia. Regarding student assessment method of preceptors for Medical Laboratory Science clinical practicum education programs in Ethiopia, almost 42.7% of the study participants were within a degree of competency of being not capable, beginner and advanced beginner level of scale, which requires high-level attention on the policy makers.

The summation average percent of the preceptors were not capable, beginner and advance beginner level of competency regarding the clinical practicum teaching quality improvement and advocacy of preceptors for Medical Laboratory Science clinical practicum education programs in Ethiopia were 42.9%, which implies a high demand of capacity building program to escalate to the recommended standard. Thus, we highly recommend that interventions should be given in areas where most of the need assessment participants indicated being beginners or advanced beginners. We also recommend the best priority area to be individual oral interview/exam/Viva which is scored beginner by N 37/304 (12.2%).

It is better to create awareness about preceptors and good working practice by teaching each other in the form of continual professional development (CPD), browsing websites and reading books. Moreover, take an action using the available information.

We recommend designing the performance interventions in the form of training by including the following modules: communication skills for effective preceptor ship, students assessment and feedback, teaching and instruction strategies, planning for clinical practicum learning and principles of learning and teaching in practical areas.

Furthermore, the finding of this study would be further strengthening by including all the regions which were not included in this study: Afar, Somali, Benishangul, Gambela and Tigray. And also, further studies from a wider geographical perspective with more representative samples are needed which could use open-ended questions, in-depth interviews or focus group discussion for adequate assessment of training need for preceptors.

## Supporting information

S1 Data Survey of preceptors training needs assessment for medical laboratory professional clinical education programs in Ethiopia. (PDF)

## Abbreviations

CPD: Continual Professional Development
EMLA: Ethiopian Medical Laboratory Association
HWIP: Health Workforce Improvement Program
