# Supplementary material for: Preceptors training need assessment for medical laboratory professional clinical education programs in Ethiopia
Source: PLoS One. 2022 Oct 6;17(10):e0275533. doi: 10.1371/journal.pone.0275533 (PMC9536571; doi:10.1371/journal.pone.0275533)
Supplement: S1 Data — (PDF) [file pone.0275533.s001.pdf]

Preceptors Data Collection Tools

Preceptors training need assessment for medical laboratory professional Clinical Education programs in Ethiopia

Information sheet

Dear participants,

The health workforce improvement program and Ethiopian Medical Laboratory Association are conducting preceptor education need assessment to assess clinical teaching, assessment, communication and advocacy skill of preceptors. The assessment includes data related to learning in the clinical/practicum area, communication and collaboration, student assessment and clinical teaching quality improvement and advocacy. The educational need assessment will help Health Workforce Improvement program (HWIP), Ethiopian Medical Laboratory Association and Higher Education Institutions (HEIs) to identify academic training needs of preceptor and design need based performance improvement trainings that will help in the creation of confident, independent, knowledgeable, responsible, and skilled preceptors.

This study has no risk on you and no payment charged for participating in this study. It only takes your time. The information that you are providing will be kept confidential. There is no personal identifier filled in the data collection tool. The findings of the study will be general for the study community and will not reflect anything particular of the individual person or its institution. No reference will be made in oral or written reports that could link participants to the research. Participation in this study is fully voluntary. You have the right to declare to participate or not in this study. If you decide to participate, you have the right to withdraw from the study at any time and this will not label you for any loss of benefits which you otherwise entitled. You do not have to answer any question that you do does not want to answer.

Contact address:

For any queries please don't hesitate to contact Mr Abebe Edao reached at Phone no 0913855657 or Email: abebe.edao@aau.edu.et.

Informed consent

Here under signing this document, I am giving my consent to participate in the study entitled as Preceptor educational training need assessment. I have been informed that the purpose of this study is to assess Preceptor's pedagogical competency. I have understood that involvement in this study is completely voluntarily. I have been told that my answers to the questions will not be given to anyone else and no reports of this study ever identify me in any way. I have also been informed that my participation or non-participation or my refusal to answer questions will have no effect on me. I understood that participation in this study does not involve risks. I understood that Mr/Mrs…………… is the contact person if I have questions about the study or about my rights as a study participant.

Respondent's signature___________________

Interviewer's name______________________ Signature ____________Date__________

Instruction:

The expected time you take to respond to the questions will take 15 minutes. The tool is divided under the following parts or sections:

- Part I: Background Information
- Part II: Learning in the clinical/ practicum area
- Part III: Communication, Collaboration and Partnership
- Part IV: Student Assessment
- Part V: Clinical Teaching Quality Improvement and Advocacy
- Part VI: Challenges and solutions
- Part VII: Specific training needs

**PART I. Background Information**		
Date of survey (Day/Month/Year):	// 2021 GC	
Q1. Region:		
Q2. Zone/Woreda:		
Q3. Name of health facility:		
Q4. Identify the type of training institution where the students came for clinical laboratory practice	Teaching hospitals<br>Referral Hospitals<br>General Hospitals<br>Primary Hospitals<br>Health centers<br>Private Teaching hospitals<br>Government Research Laboratories<br>Private research/advanced diagnostic laboratories<br>Others (specify)	1<br>2<br>3<br>4<br>5<br>6<br>7<br>8<br>9
	Please specify the name of the training institution___________	
Q5. Sex	Female ……………………………….……..……….1<br>Male …………………………….……….………..2	
Q6. Age	__________________Years	

Q7 . Level of Education (Diploma, BSc, MD, MSc, PhD…)	___________________
Q8. Please mention your specific field of study	____________________________
Q9. Year of experience	_____________________Years
Q10. Have you received clinical/practicum teaching skills training in the past two years?	Yes ……………………………………………..1<br>No ……………………………………………….2<br>If yes please specify _________________________
Q.11 Year of experience in preceptor-ship/student teaching	___________________ Years
Q12, Do you know your role and responsibilities as a trainer/preceptor?	Yes ……………………………………………1<br>No --2
Q13. What type of capacity building mode of delivery do you prefer? (Select one best option only)	1. Hands on training<br>2. Virtual<br>3. If others, please specify…………………
Q14. Do you have access to computer	Yes ……………………………………………1<br>No --2
Q15. Do you have access to internet?	Yes ……………………………………………1<br>No --2

Instruction:

Please **encircle** your "degree of competency-as indicated in the left side" and "frequency of applying- as indicated in the right side" the skills below and respond (write) the open ended questions on the basis of your actual practice.
Remember the following 4 points could be considered as a simple competency scales from the lowest "not capable" to the highest "proficient" in order.

- **1 (Not capable):** requires training and supervision before performing the task
- **2 (Beginner):** Able to perform the task under direct or indirect supervision
- **3 (Competent):** Able to perform the task routinely without the need for supervision
- **4 (Proficient):** Able to perform the task routinely, even in complex contexts, and can train and supervise other teachers/ preceptors

Skill set/ area	What is your perceived level of competency on the skill?				How frequently do you apply the skill?				
**PART II. Learning in the practical teaching area**	Not capable (1)	Beginner (2)	Competent (3)	Proficient (4)	Never (1)	Rarely (2)	Monthly (3)	Weekly (4)	Daily (5)
1. Develop clear and measurable learning objectives for practicum/clinical sessions	❶	❷	❸	❹	❶	❷	❸	❹	❺
2. Develop plan for practicum/clinical teaching sessions	❶	❷	❸	❹	❶	❷	❸	❹	❺
3. Obtain informed consent for student involvement in actual practice	❶	❷	❸	❹	❶	❷	❸	❹	❺
4. Select appropriate teaching and learning materials and resources that are matched to the learning outcomes.	❶	❷	❸	❹	❶	❷	❸	❹	❺

5. Apply different hands-on teaching strategies/ methods that are matched to the learning outcomes									
5.1 Demonstration	❶	❷	❸	❹	❶	❷	❸	❹	❺
5.2 Coaching	❶	❷	❸	❹	❶	❷	❸	❹	❺
5.3 Case-based teaching/ Case study	❶	❷	❸	❹	❶	❷	❸	❹	❺
5.4 Role play	❶	❷	❸	❹	❶	❷	❸	❹	❺
5.5 Group discussion	❶	❷	❸	❹	❶	❷	❸	❹	❺
5.6 Seminar presentations	❶	❷	❸	❹	❶	❷	❸	❹	❺
5.7 Industry attachment	❶	❷	❸	❹	❶	❷	❸	❹	❺
5.8 Laboratory practice	❶	❷	❸	❹	❶	❷	❸	❹	❺
5.9 Side lab.	❶	❷	❸	❹	❶	❷	❸	❹	❺
5.10 Community based training	❶	❷	❸	❹	❶	❷	❸	❹	❺
6. Create opportunities for learners to develop critical thinking and reasoning skills	❶	❷	❸	❹	❶	❷	❸	❹	❺
7. Create and maintain a safe practical teaching environment for the students that is free from physical, psychological or any other form of harm	❶	❷	❸	❹	❶	❷	❸	❹	❺
8. Engage students in critical and constructive self-assessment skill and peer feedback	❶	❷	❸	❹	❶	❷	❸	❹	❺
9. Apply principles of gender responsive pedagogy in:									
9.1. Developing plan for clinical practicum teaching	❶	❷	❸	❹	❶	❷	❸	❹	❺
9.2. Facilitating clinical practicum session	❶	❷	❸	❹	❶	❷	❸	❹	❺
9.3. Engage in effective guidance and counseling that help learners	❶	❷	❸	❹	❶	❷	❸	❹	❺

meet their learning goals									
9.4. Prevent and respond gender based violence	❶	❷	❸	❹	❶	❷	❸	❹	❺
10. Demonstrate the principles of effective delegation and supervision to students	❶	❷	❸	❹	❶	❷	❸	❹	❺
11. Use open educational (updated guidelines, protocols, research findings, digital learning platforms, etc) resources effectively	❶	❷	❸	❹	❶	❷	❸	❹	❺
**PART III. Communication, Collaboration and Partnership**	**Not capable (1)**	**Beginner (2)**	**Competent (3)**	**Proficient (4)**	**Never (1)**	**Rarely (2)**	**Monthly (3)**	**Weekly (4)**	**Daily (5)**
12. Communicate best practice in practical education with peers, students and other stakeholders.	❶	❷	❸	❹	❶	❷	❸	❹	❺
13. Foster teamwork and collaboration between academic and practical environment	❶	❷	❸	❹	❶	❷	❸	❹	❺
14. Build and maintain collegial relationships and collaborations with multidisciplinary staff in the practical practicum teaching environment	❶	❷	❸	❹	❶	❷	❸	❹	❺
15. Use oral, written and electronic communication in practical teaching in order to achieve learner outcomes.	❶	❷	❸	❹	❶	❷	❸	❹	❺
16. Teach students on how to address patients with special need	❶	❷	❸	❹	❶	❷	❸	❹	❺
17. Teach students how to provide health education to patients and their families/ caregiver.	❶	❷	❸	❹	❶	❷	❸	❹	❺

**PART IV. Student Assessment**	**Not capable (1)**	**Beginner (2)**	**Competent (3)**	**Proficient (4)**	**Never (1)**	**Rarely (2)**	**Monthly (3)**	**Weekly (4)**	**Daily (5)**
18. Select and use assessment methods that are matched with the learning outcomes	➊	➋	➌	➍	➊	➋	➌	➍	➎
19. Employ different practical teaching assessment methods and tools									
19.1. Workplace based assessments (e.g. Directly observed procedure, Practical clinical Evaluation, Case based discussion)	➊	➋	➌	➍	➊	➋	➌	➍	➎
19.2. Individual oral interview/exam/Viva	➊	➋	➌	➍	➊	➋	➌	➍	➎
19.3. Objectively structured practical exams (OSPEs)	➊	➋	➌	➍	➊	➋	➌	➍	➎
19.4. Portfolio/report	➊	➋	➌	➍	➊	➋	➌	➍	➎
19.5. Performance/ procedural checklist	➊	➋	➌	➍	➊	➋	➌	➍	➎
20. Provide timely constructive feedback to students	➊	➋	➌	➍	➊	➋	➌	➍	➎
21. Maintain accurate records of student progress	➊	➋	➌	➍	➊	➋	➌	➍	➎
22. Use assessment data to enhance the practical teaching-learning process	➊	➋	➌	➍	➊	➋	➌	➍	➎

**PART V. Clinical practicum teaching quality improvement and Advocacy**	**Not capable (1)**	**Beginner (2)**	**Competent (3)**	**Proficient (4)**	**Never (1)**	**Rarely (2)**	**Monthly (3)**	**Weekly (4)**	**Daily (5)**
23. Practice regular self-evaluation, reflection and receive feedback from peers and students	➊	➋	➌	➍	➊	➋	➌	➍	➎
24. Maintain current self-development	➊	➋	➌	➍	➊	➋	➌	➍	➎

activities and a curriculum vitae and portfolio.									
25. Contribute to the quality assurance processes of clinical practicum teaching	❶	❷	❸	❹	❶	❷	❸	❹	❺
26. Adherence to policy and procedures	❶	❷	❸	❹	❶	❷	❸	❹	❺
27. Evaluate clinical practicum education program to ensure attainment of national program standards	❶	❷	❸	❹	❶	❷	❸	❹	❺
28. Participate in quality reviews to assess strengths and weaknesses of the program	❶	❷	❸	❹	❶	❷	❸	❹	❺
29. Use of a variety of advocacy strategies to promote clinical practicum education and practice.	❶	❷	❸	❹	❶	❷	❸	❹	❺
30. Create and maintain clinical practicum partnership that support educational goals	❶	❷	❸	❹	❶	❷	❸	❹	❺
**PART VI. Challenges and solutions**									
31. List the challenges in the implementation of the above practical skill areas	______________________<br>______________________<br>______________________								
32. Please list experience in applying principles of gender responsive pedagogy, if any…………									
**PART VII: Specific training needs**									
33. List the roles you assume as preceptor in your	1. ______________________								

organization	2. ______<br>3. ______<br>4. ______<br>5. ______
34. In relation to the clinical practicum teaching skills, please specify the areas of your job in which you would like to receive further training or instruction. List these in order of importance:	1.<br>2.<br>3.<br>4.<br>5.
35. For the above skill gap, what deliverable modalities do you prefer for your learning? List these in order of preference:	1.<br>2.<br>3.<br>4.

Do you have any additional comments or suggestions?

______

______

______
